# Endothelial Endothelin Receptor A Expression Is Associated With Podocyte Injury and Oxidative Stress in Patients With Focal Segmental Glomerulosclerosis

**DOI:** 10.1016/j.ekir.2021.04.013

**Published:** 2021-04-22

**Authors:** Nina A. van de Lest, Aimée E. Bakker, Kyra L. Dijkstra, Malu Zandbergen, Sharon A.C. Heemskerk, Ron Wolterbeek, Jan A. Bruijn, Marion Scharpfenecker

**Affiliations:** 1 Department of Pathology, Leiden University Medical Center, Leiden, Netherlands; 2 Medical Statistics, Department of Biomedical Data Sciences, Leiden University Medical Center, Leiden, Netherlands

**Keywords:** crosstalk, endothelin receptor A, focal segmental glomerulosclerosis, glomerular endothelial cells, oxidative stress, podocytes

## Abstract

**Introduction:**

The podocyte is thought to be the mainly affected cell type in focal segmental glomerulosclerosis (FSGS). However, recent studies have also indicated a role for glomerular endothelial cells and podocyte−endothelial crosstalk in FSGS development. An experimental model for podocyte injury showed that increased endothelin-1 (ET-1) signaling between podocytes and endothelial cells induces endothelial oxidative stress and subsequent podocyte loss. In the current study, we investigated endothelial endothelin receptor A (ET_A_R) expression in patients with FSGS and its association with podocyte injury and glomerular oxidative stress.

**Methods:**

We selected 39 biopsy samples of patients with FSGS and 8 healthy control subjects, and stained them for ET_A_R, nephrin and 8-oxo-guanine, a DNA lesion caused by oxidative damage. Glomeruli with ET_A_R-positive endothelium and with nephrin loss were scored, and the 8-oxo-guanine−positive glomerular area was measured.

**Results:**

The mean percentage of glomeruli with ET_A_R-positive endothelial cells in patients with FSGS was higher compared to that in healthy control subjects (52% vs. 7%; *P* < 0.001). The presence of glomerular ET_A_R-positive endothelium was strongly associated with nephrin loss both on the biopsy level (rho = 0.47; *P* < 0.01), as on the level of individual glomeruli (odds ratio = 2.0; *P* < 0.001). Moreover, glomeruli with ET_A_R-positive endothelium showed more 8-oxo-guanine−positive staining (1.9% vs. 2.4%; *P* = 0.037). Finally, 8-oxo-guanine positivity in glomeruli was associated with increased levels of proteinuria.

**Conclusion:**

Taking together our findings, we show that ET_A_R is increased in glomerular endothelial cells of patients with FSGS and associated with podocyte damage and glomerular oxidative stress. These findings support the hypothesis that ET-1 signaling in glomerular endothelial cells contributes to disease development in patients with FSGS.

## Graphical abstract

**Figure:**
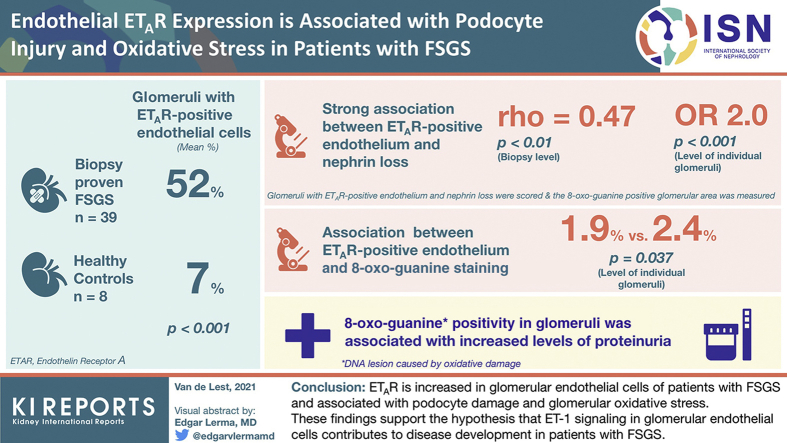

See Commentary on Page 1758

Focal segmental glomerulosclerosis (FSGS) describes a pattern of glomerular damage that results from various types of podocyte injury.^1^ The primary form of FSGS presents clinically with heavy proteinuria and nephrotic syndrome. Initial podocyte injury is thought to be the main driver of this disease. Therefore, research investigating the development and pathogenesis of FSGS has predominantly focused on the role of the podocyte.

Yet, an increasing body of evidence suggests a role for the glomerular endothelium in the pathogenesis of FSGS as well. Structural alterations in endothelial cells have been shown in both primary and secondary FSGS.^2^, ^3^, ^4^, ^5^, ^6^ These findings are supported by several experimental studies that provide evidence for a role of the glomerular endothelium in FSGS.^7^^,^^8^ Moreover, in an experimental model of podocyte injury induced by transforming growth factor−β (TGF-β) signaling, Daehn *et al.* show that crosstalk between endothelial cells and podocytes plays an important role in the development of proteinuria and glomerulosclerosis. The underlying mechanism was based mainly on altered endothelin-1 (ET-1) signaling between podocytes and endothelial cells.^9^^,^^10^

Endothelin-1 is a small peptide that was originally thought of as a potent vasoconstrictor. However, it has become evident that ET-1 exerts many different effects throughout the body via the endothelin receptors A and B (ET_A_R and ET_B_R, respectively).^11^ In the healthy kidney, ET_A_R and ET_B_R are expressed in different regions: ET_A_R is mainly localized in the smooth muscle cells of the renal vasculature, and ET_B_R is abundantly present in the renal medulla as well as in endothelial cells and glomeruli.^12^^,^^13^ ET-1 is implicated in the progression of chronic kidney disease (CKD) by promoting tubulointerstitial inflammation and fibrosis and glomerular injury.^14^ The influence of ET-1 on the progression of CKD is thought to be the result of an imbalance of ET_A_R over ET_B_R activation. Increased levels of serum and urinary ET-1 in patients with FSGS and the beneficial effects of ET_A_R blockers in experimental age-associated FSGS suggest that the endothelin system is involved in the pathogenesis of FSGS as well.^15^^,^^16^

Previous studies have suggested that ET-1-ET_A_R signaling in podocytes causes podocyte damage, which would promote FSGS development. In contrast, the above-mentioned study by Daehn *et al.* shows that podocyte-derived ET-1 activates ET_A_R expressed on endothelial cells rather than on podocytes, subsequently leading to podocyte apoptosis.^9^ Moreover, this study also demonstrates that ET-1 signaling causes accumulation of 8-oxo-guanine (8-oxoG), a DNA lesion induced by oxidative damage, in mitochondrial DNA of glomerular endothelial cells, and that this accumulation promotes the progression of podocyte injury. These results suggest that altered podocyte−endothelial crosstalk via ET-1 and ET_A_R contributes to the progression of FSGS.

Involvement of both the endothelin system and oxidative stress in the development of FSGS have been suggested earlier.^11^^,^^17^, ^18^, ^19^, ^20^ Yet, these mechanisms were not previously linked to endothelial changes and altered podocyte−endothelial crosstalk. Although *in vivo* studies have demonstrated that targeting oxidative stress can ameliorate the FSGS phenotype, it has not been confirmed whether these effects are due to reduced oxidative stress levels in endothelial cells or podocytes.^17^, ^18^, ^19^, ^20^ Glomerular endothelial cells are generally thought to primarily express ET_B_R,^12^ and expression of ET_A_R has been reported only under specific pathological circumstances.^9^^,^^10^^,^^21^^,^^22^ Daehn *et al.* clearly showed that endothelial ET_A_R expression was increased in their murine model of podocyte injury. The identification of ET_A_R in endothelial cells of patients with FSGS and its involvement in podocyte−endothelial crosstalk would shed light onto a potential new mechanism of action of the endothelin system in the development of human FSGS.

In the current study, we therefore analyzed ET-1 signaling in glomerular endothelial cells by determining ET_A_R expression. In addition, we assessed whether increased ET_A_R expression was associated with podocyte damage and increased glomerular oxidative stress. We show, for the first time, that ET_A_R expression is upregulated in glomerular endothelial cells of patients with FSGS, and that increased expression is associated with podocyte damage and glomerular oxidative stress. These data suggest that ET-1 signaling is increased in glomerular endothelial cells, and therefore support the hypothesis of altered podocyte−endothelial crosstalk via the endothelin system in patients with FSGS.

## Materials and Methods

### Biopsy Sample Collection

Biopsy or kidney samples from patients with FSGS were obtained from the archives of the Department of Pathology at the Leiden University Medical Center; the biopsies were performed between 1985 and 2017. We selected patients with FSGS for which the underlying pathology was unknown. Cases of FSGS based on renal comorbidity (other than minimal change disease), virus-induced FSGS, or medication-induced FSGS were excluded. Transplant biopsy samples with FSGS were excluded as well. We included patients with hypertension, atherosclerosis, and/or obesity of which it was unclear whether these clinical findings caused FSGS. Biopsy samples with insufficient material for analysis (<5 glomeruli) were excluded. Finally, we analyzed 39 biopsy samples. As control samples, we used 8 Eurotransplant kidney sections obtained from deceased donors that were not suitable for transplantation for technical reasons.

### Immunohistochemistry and Immunofluorescence

Sequential 4-μm-thick sections of paraffin-embedded kidney or biopsy samples were stained for ET_A_R, nephrin, 8-oxoG and periodic acid−Schiff (PAS). Stainings were performed using a rabbit anti-human ET_A_R primary antibody (1:400; Alomone, Jerusalem, Israel), a rabbit anti-human nephrin primary antibody (1:750; Abcam, Cambridge, UK), or a mouse IgG2b anti-human 8-OHdG (8-oxoG) anti-body (1:4800; Santa Cruz, Dallas, TX). 8-oxoG−Stained sections were pretreated with RNase.^23^^,^^24^ The appropriate isotype control fraction at the same concentration as the primary antibody was used as negative control. An EnVision horseradish peroxidase‒conjugated antibody (Dako, Glostrup, Denmark) was used as the secondary antibody, and staining was visualized using diaminobenzidine. Sections were counterstained with hematoxylin. The sections were visualized using a Philips Ultra-Fast Scanner (Philips, Amsterdam, Netherlands) for immunohistochemical analysis. A blocking peptide experiment was performed to confirm ET_A_R antibody specificity (Supplementary Figure S1).

Immunofluorescence was performed on 4-μm-thick sections of paraffin-embedded kidney or biopsy samples as well. ET_A_R staining was performed using the same antibody as for immunohistochemistry in a 1:100 dilution. CD31 staining was performed using a mouse IgG1 anti-human CD31 antibody (1:200, Dako). 8-oxoG Double staining was performed using the same primary antibody as for immunohistochemistry in a 1:500 dilution. von Willebrand factor (vWF) and Wilms tumor 1 (WT1) staining were performed using a goat anti-human vWF antibody (1:400, Affinity Biologicals, Ancaster, ON, Canada) and a rabbit anti-human WT1 antibody (1:400, Santa Cruz). Alexa Fluor 488 and 546 (Thermo Fisher Scientific, Waltham, MA) were used as secondary antibodies, and sections were covered with Prolong Gold containing DAPI (Thermo Fisher Scientific). Detailed examples of antibody controls can be found in Supplementary Figures S2 and S3. Double stainings were performed on 2 to 3 cases with FSGS. Images were captured using a Zeiss confocal laser scanning microscope with ZEN software (Zeiss, Oberkochen, Germany).

### Staining Evaluation

All assessable glomeruli in a section were used for immunohistochemistry analysis. ET_A_R staining was scored by 2 independent observers. A glomerulus was considered positive for endothelial ET_A_R staining when circumferential staining in at least 1 capillary loop was present. An adjacent PAS-stained section was used to assess morphology. A consensus meeting was held for those glomeruli with interobserver variability. The average difference in ET_A_R positivity score between observers was seen in 5.2% of glomeruli. Nephrin loss was defined as >10% interruption in the circumferential nephrin staining surrounding the glomerular tuft. For detailed descriptions and representative examples, please see our previous publication.^25^ The adjacent PAS-stained section was used to identify nephrin loss in areas of glomerulosclerosis. The 8-oxoG−positive staining area was measured using ImageJ software and presented as percentage positive area of the total glomerular tuft area. Summary data show the percentage of glomeruli in a biopsy sample with ET_A_R-positive endothelium or nephrin loss or the mean 8-oxoG−positive area of all glomeruli in a biopsy sample. The cut-off for 8-oxoG positivity for a biopsy sample was based on the upper whisker limit excluding outliers of the control samples. The upper limit was 1.23%, which was averaged to a cut-off of 1.5%. Colocalization of ET_A_R, nephrin and 8-oxoG in the same glomerulus was assessed by identifying the same glomerulus in sequential adjacent sections of the same biopsy.

### Statistical Analysis

Data were analyzed using IBM SPSS Statistics, Version 25.0 (IBM Corp., Armonk, NY). Dichotomous data were analyzed using the χ^2^/Fisher exact test. Continuous data were analyzed using the Student *t* test or Mann−Whitney *U* test. Correlations were analyzed using Pearson r or Spearman rho correlation coefficient. Summary data are presented as the mean ± SD, as the median with interquartile range, or as a number with a percentage. Binary outcomes for individual glomeruli were used to determine the probability of nephrin loss for glomeruli with ET_A_R-positive endothelium. To take into account the correlation among glomeruli in the same patient, we used logistic regression within generalized estimating equation (GEE) analysis. The association between 8-oxoG−positive area and ET_A_R-positive endothelium or nephrin loss within glomeruli was calculated using linear mixed model analysis, thus taking into account observations clustered within patients. The α level was set to 0.05.

### Ethics

Tissue samples were collected during routine patient care. For medical research purposes, the tissues were coded and then handled anonymously in accordance with the Dutch National Ethics Guidelines (Code for Proper Secondary Use of Human Tissue) and the Declaration of Helsinki.

## Results

### ET_A_R Expression in Glomerular Endothelial Cells of Patients With FSGS

**Figure 1:**
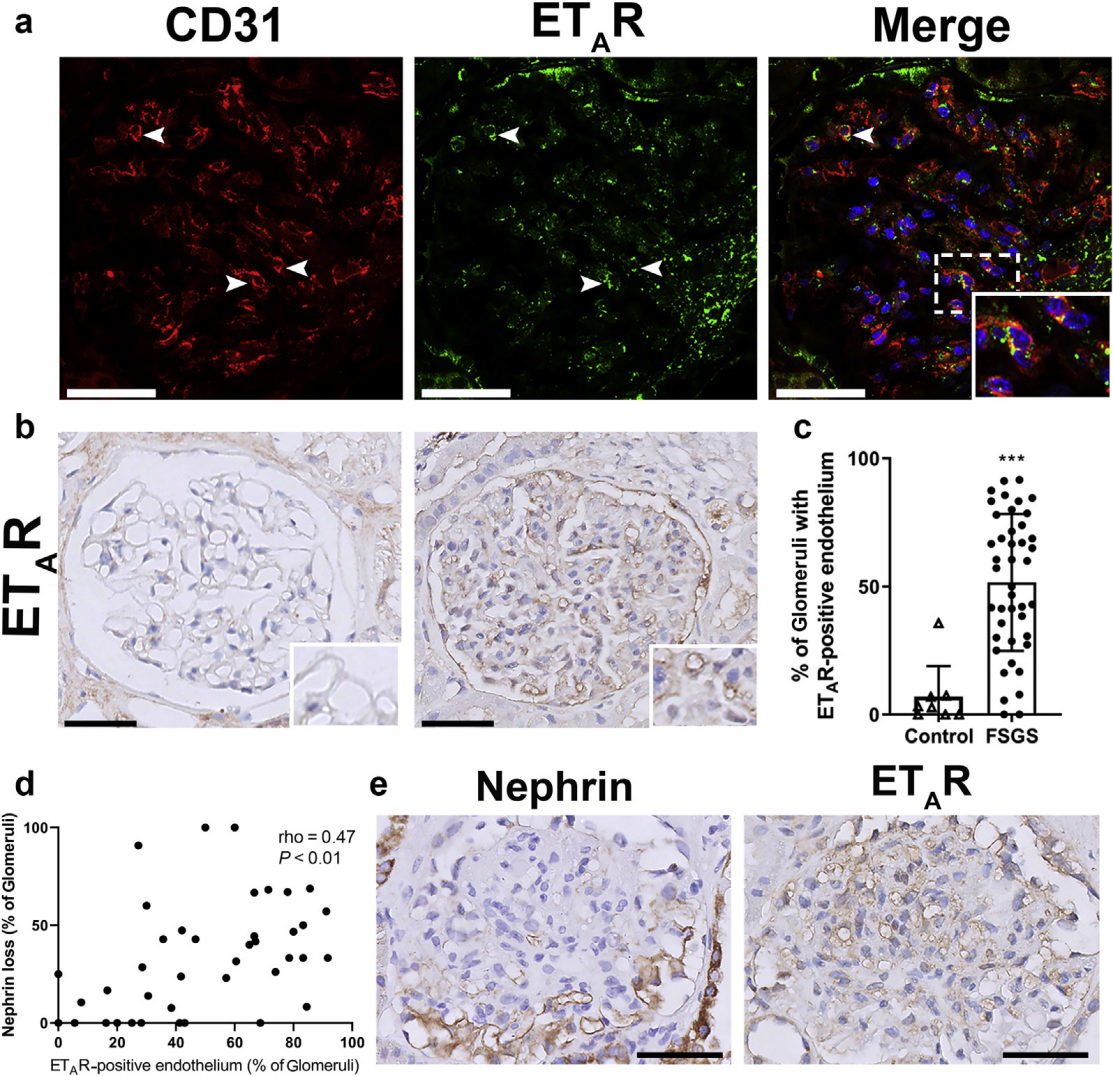
Increased endothelial endothelin receptor A (ET_A_R) expression in glomeruli of patients with focal segmental glomerulosclerosis (FSGS). (a) ET_A_R staining (green) colocalizes with endothelial CD31 (red), indicating that ET_A_R is expressed on glomerular endothelial cells (enlarged in inset). (b) Representative examples of glomerular ET_A_R expression in control kidneys (left) and biopsy samples with FSGS (right). Insets highlight capillaries without ET_A_R-positive staining (control) and with ET_A_R-positive staining (FSGS). (c) Quantification of the percentage of glomeruli with ET_A_R-positive endothelium. The mean percentage of glomeruli with ET_A_R-positive endothelial cells per patient was higher in patients with FSGS compared to healthy control subjects (52% vs. 7%; *P* < 0.001). (d) The percentage of glomeruli with ET_A_R-positive endothelium per patient correlated with the percentage of glomeruli with nephrin loss in patients with FSGS. (e) Nephrin loss and ET_A_R-positive endothelium colocalized within the same glomeruli. Areas of nephrin loss were positive for an endothelial pattern of ET_A_R, whereas areas with normal linear nephrin expression did not show ET_A_R positivity. Analyzing all individual glomeruli for nephrin loss and ET_A_R expression in sequential sections of the same glomerulus showed that the odds of having nephrin loss was higher for glomeruli with ET_A_R-positive endothelium compared to ET_A_R-negative glomeruli (OR = 2.0; *P* < 0.001). Bars = 50 μm.

To investigate whether increased ET-1 signaling via the ET_A_R could be present in glomerular endothelial cells in FSGS, we collected biopsy samples of patients with FSGS and stained them for ET_A_R. First, we assessed whether ET_A_R-positive staining was present in endothelial cells. Therefore, we performed an immunofluorescent double staining of ET_A_R with CD31. Figure 1a shows colocalization of CD31 (red) with ET_A_R (green) in a glomerulus of a patient with FSGS, indicating that ET_A_R was expressed in glomerular endothelial cells (yellow). Next, we stained 39 biopsy samples of patients with FSGS and 8 control subjects for ET_A_R with immunohistochemistry and assessed the presence of an endothelial staining pattern in glomeruli. The clinical characteristics of these patients are provided in Table 1. Demographic characteristics of control subjects can be found in Supplementary Table S1. Figure 1b shows a representative example of an ET_A_R-negative glomerulus in a healthy control subject (left) and a representative example of a glomerulus with an endothelial staining pattern of ET_A_R of a patient with FSGS (right). Summary data of the mean percentage of glomeruli with ET_A_R-positive endothelial staining can be found in Figure 1c. The percentage of glomeruli with ET_A_R staining in endothelial cells was significantly higher in patients with FSGS compared to healthy control subjects (52% vs. 7%; *P* < 0.001). ET_A_R positivity in endothelial cells was associated with a higher age at the time of biopsy (R = 0.33; *P* = 0.044), but did not correlate with other characteristics (Table 2 and Supplementary Table S2).

**Table 1:** Clinical characteristics of patients with FSGS at time of biopsy (n = 39)

Characteristic	Value
Age, yr, mean ± SD	36 ± 21
Sex, male, n (%)	20 (51)
Proteinuria, g/24 h, mean ± SD	7.6 ± 6.5
eGFR, ml/min per 1.73 m^2^, mean ± SD	77 ± 39
FSGS variant, n (%)	
NOS	27 (69)
Perihilar	1 (3)
Cellular	5 (13)
Tip	3 (8)
Collapsing	3 (8)
Medication, n (%)	
None	2 (5)
Conservative	12 (31)
Prednisone	7 (18)
Other immunosuppressive agents	5 (13)
Unknown	13 (33)

eGFR, estimated glomerular filtration rate; FSGS, focal segmental glomerulosclerosis; NOS, not otherwise specified.

**Table 2:** Correlations between the percentage of glomeruli with ET_A_R-positive endothelium and clinical characteristics

	Correlation coefficient^a^	*P* value
Age	0.33	0.044
Proteinuria	0.02	0.94
eGFR	−0.185	0.318

eGFR, estimated glomerular filtration rate; ET_A_R, endothelial endothelin receptor A.

a Pearson correlation.

### Increased ET_A_R Expression and the Association With Podocyte Injury

Next, we assessed whether increased ET_A_R expression in glomerular endothelial cells of patients with FSGS was associated with podocyte damage. In a previous publication, we demonstrated that segmental nephrin loss occurs early in the process of podocyte damage, while other podocyte markers are still present.^25^ Thus, we used nephrin loss as a marker for podocyte injury. We stained the biopsy samples of patients with FSGS for nephrin and determined the presence of nephrin loss for each glomerulus. The mean percentage of glomeruli with nephrin loss was then calculated for each biopsy. Figure 1d shows that the percentage of glomeruli with ET_A_R-positive endothelial staining and the percentage of glomeruli with nephrin loss in a biopsy sample were significantly correlated (rho = 0.47, *P* < 0.01). Because nephrin loss can occur because of glomerulosclerotic lesions, we also calculated the percentage of glomeruli with nephrin loss when excluding nephrin loss due to glomerulosclerotic lesions. Supplementary Figure S4 shows that, with this correction, nephrin loss and ET_A_R positivity were still correlated (rho = 0.38, *P* < 0.05).

In addition, we determined the association between nephrin loss and endothelial ET_A_R positivity within individual glomeruli. In Figure 1e, adjacent sections of the same glomerulus are shown. In the area of nephrin loss, ET_A_R was abundantly present. In contrast, no ET_A_R was present in the area that was still positive for nephrin. When analyzing all glomeruli, the odds of having nephrin loss was 2 times higher for glomeruli with ET_A_R-positive endothelium (odds ratio [OR] = 2.0, confidence interval [CI] =1.4−2.7; *P* < 0.001), indicating that there was also a correlation between nephrin loss and ET_A_R positivity in individual glomeruli.

**Figure 2:**
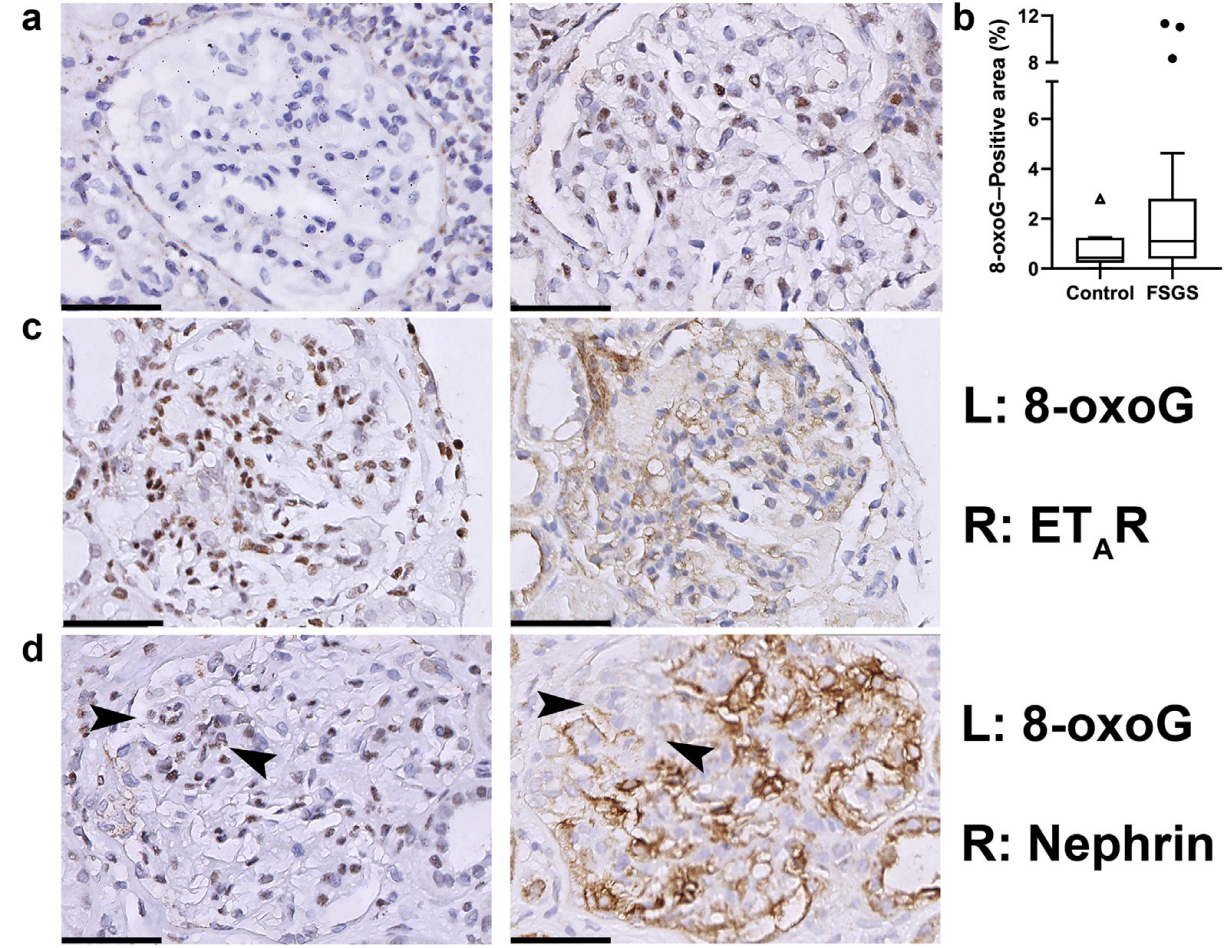
8-oxo-guanine (8-oxoG) Staining in glomeruli of patients with focal segmental glomerulosclerosis (FSGS) indicates increased oxidative stress and correlates with ET_A_R expression. (a) Representative examples of 8-oxoG staining in (left) a transplant control kidney sample and (right) a biopsy sample of a patient with FSGS. (b) Quantification of glomerular 8-oxoG as measured by ImageJ. Graph shows the summary data of the mean 8-oxoG−positive area per patient. Although the median 8-oxoG expression was not different in patients with FSGS compared to controls, there was a subset of FSGS patients with increased 8-oxoG levels (as indicated by the higher spread in the upper quadrants). (c) In patients with FSGS, glomeruli with ET_A_R-positive endothelium showed more 8-oxoG−positive staining. (d) Occasionally, we observed colocalization of 8-oxoG−positive cells within areas of nephrin loss (arrowheads). Bars = 50 μm.

### Oxidative Stress in Glomeruli of Patients With FSGS and the Association With ET_A_R

**Figure 3:**
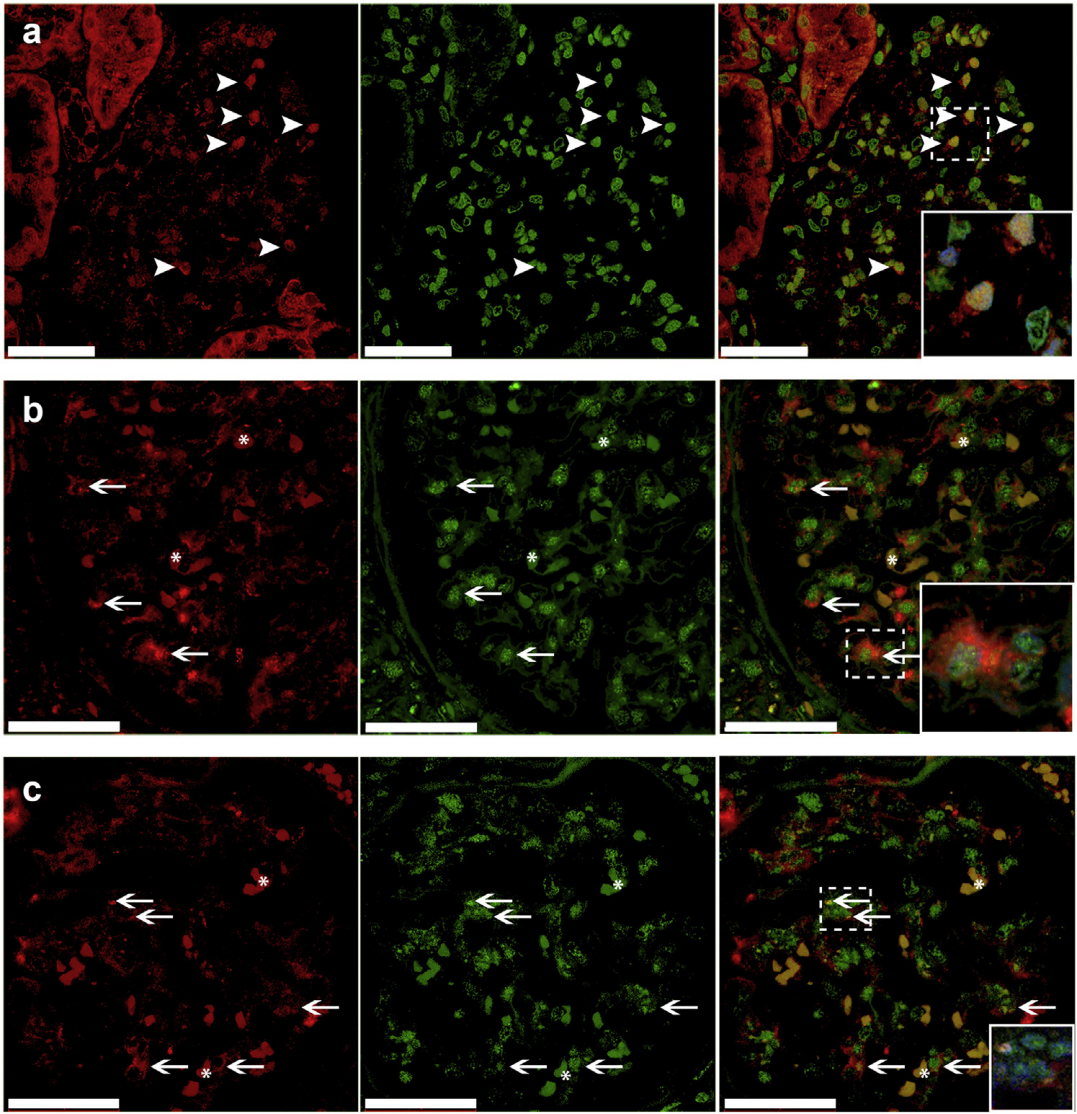
8-oxoG−Positive staining in podocytes and endothelial cells of patients with FSGS. (a) Wilms tumor 1−positive staining (red) colocalized with 8-oxoG−positive staining (green) in glomeruli of patients with FSGS, indicating that oxidative stress levels are increased in podocytes (arrowheads) (enlarged in inset; with DAPI). (b, c) von Willebrand factor−positive staining (red) also colocalized with 8-oxoG (green) positive staining, indicating that endothelial cells also had increased oxidative stress levels (arrows). Part b shows nuclear 8-oxoG staining enclosed by cytoplasmic von Willebrand Factor staining (enlarged in inset; with DAPI). Part c shows cytoplasmic 8-oxoG staining in yellow, colocalizing with cytoplasmic von Willebrand factor staining (enlarged in inset; with DAPI). Bars = 50 μm. Asterisks in parts b and c illustrate examples of autofluorescence of erythrocytes present in glomerular capillaries, which is commonly observed.^27^^,^^28^

Next, we set out to determine oxidative stress levels in our biopsy cohort of patients with FSGS. We used 8-oxoG as a marker for oxidative stress. 8-oxoG Is one of the major DNA lesions generated by reactive oxygen species (ROS) under oxidative stress. It can both accumulate in nuclear and mitochondrial DNA and is often used as a stable marker for oxidative stress. Glomerular 8-oxoG positivity has been observed in experimental models of FSGS.^9^^,^^26^ In Figure 2a, example images of a glomerulus negative for 8-oxoG and of a glomerulus with abundant 8-oxoG staining are shown. In contrast to what was expected, the median 8-oxoG−immunopositive area of the glomerular tuft in patients with FSGS was not different compared to that of control subjects (median = 1.0%, interquartile range [IQR] = 0.40%−2.7% vs. median = 0.43%, IQR = 0.23%−1.2%, respectively; *P* = 0.19) (Figure 2b). Within biopsy samples, the mean 8-oxoG−positive area did not correlate with the percentage of glomeruli with ET_A_R-positive endothelial staining or the percentage of glomeruli with nephrin loss. However, when analyzing individual glomeruli, we found that glomeruli with ET_A_R-positive endothelium showed more 8-oxoG−positive staining compared to ET_A_R-negative glomeruli (1.9 vs. 2.4; *P* = 0.037) (representative picture in Figure 2c). This association was not found for 8-oxoG and nephrin loss, although we did occasionally observe clearly increased 8-oxoG staining in areas of nephrin loss (Figure 2d). We performed immunofluorescent double staining to determine whether 8-oxoG was present in glomerular endothelial cells and/or podocytes. Figure 3a shows colocalization of 8-oxoG with WT1, indicating that 8-oxoG was expressed in podocytes. Figure 3b and c show colocalization of 8-oxoG with vWF. Because most of the 8-oxoG staining was present in nuclei and because vWF is a cytoplasmic endothelial marker, 8-oxoG and vWF often did not colocalize, but the enclosure of vWF-positive staining around 8-oxoG−positive nuclei indicates 8-oxoG−positive endothelial cells (Figure 3b^27^^,^^28^). In addition, in some cells, we also observed 8-oxoG staining in the vWF-positive cytoplasm (Figure 3c^27^, ^28^).

As can be observed in Figure 2b and Supplementary Figure S5, some patients with FSGS displayed increased levels of 8-oxoG, whereas some did not show any 8-oxoG positivity. To determine whether differences existed between patients with 8-oxoG positivity and patients without, we performed an additional analysis based on a threshold of 8-oxoG positivity (mean positive area of ≥1.5%; see Material and Methods). Sixteen patients with FSGS (42%) were 8-oxoG positive, compared to 1 patient in the control group (*P* = 0.08). Table 3 shows the clinical characteristics of 8-oxoG−positive patients and 8-oxoG−negative patients. Patients who were 8-oxoG−positive had statistically significantly higher levels of proteinuria at the time of biopsy compared to patients who were 8-oxoG−negative (10.7 g/d vs. 5.3 g/d respectively; *P* < 0.05).

**Table 3:** Clinical characteristics stratified by 8-oxoG−negative and 8-oxoG−positive cases

	8-oxoG−negative (n=22)	8-oxoG−positive (n=16)	*P* value
Age, yr, mean ± SD	36 ± 23	38 ± 20	0.86^a^
Sex, male, n (%)	11 (50)	8 (50)	1.00^b^
Proteinuria, mean ± SD (g/24 h)	5.3 ± 2.8	11 ± 8.8	<0.05^a^
eGFR, mean, ± SD (ml/min per 1.73 m^2^)	78 ± 37	79 ± 45	0.94^a^
FSGS variant, n (%)			1.00^b^
NOS	14 (63)	12 (75)	
Perihilar	1 (5)	0	
Cellular	3 (14)	2 (13)	
Tip	2 (9)	1 (6)	
Collapsing	2 (9)	1 (6)	

eGFR, estimated glomerular filtration rate; FSGS, focal segmental glomerulosclerosis; NOS, not otherwise specified; 8-oxoG, 8-oxo-guanine.

a Student *t* test.

b χ^2^ or Fisher exact test.

## Discussion

In this study, we investigated whether ET_A_R expression is higher in glomerular endothelial cells of patients with FSGS and whether this is associated with increased podocyte damage and oxidative stress. To this end, we stained biopsies of patients with FSGS for ET_A_R, 8-oxoG and nephrin. We found that glomeruli of patients with FSGS show increased expression of ET_A_R in endothelial cells, which was associated with podocyte damage. Moreover, we showed that 8-oxoG positivity, a marker for oxidative stress, correlated with ET_A_R positivity and higher levels of proteinuria. These data support the hypothesis that endothelial-podocyte-crosstalk via ET-1 signaling and possibly oxidative stress is altered in the pathogenesis of FSGS.

FSGS has long been considered a disease of the podocyte. However, an increasing number of studies support a role of the glomerular endothelium in the development of FSGS as well. Associations between the severity of endothelial damage and the amount of glomerulosclerosis have been found in experimental models of FSGS.^8^^,^^10^ Moreover, pathological endothelial changes prior to structural alterations of the podocyte have both been reported in Adriamycin nephropathy and a model with constitutive overexpression of TGF-β in podocytes.^7^^,^^10^ More evidence comes from studies in patients, showing an association between endothelial injury and the development of FSGS.^3^, ^4^, ^5^^,^^29^ Recently, by using single-cell transcriptome analysis, Menon *et al.* found high glomerular endothelial activation scores in patients with FSGS, which were linked to lower proteinuria remission rates.^30^ In addition, we recently published a paper illustrating a role for complement activation in patients with FSGS.^31^ Complement activation is commonly seen in kidney diseases characterized by endothelial damage and could thus be an indicator of endothelial injury. However, despite these studies supporting a role for the glomerular endothelium in FSGS, altered endothelial−podocyte crosstalk has not been comprehensively investigated in patients with FSGS.

We now provide data suggesting that the endothelin system plays a role in endothelial−podocyte interaction in patients with FSGS. This is supported by *in vitro* and *in vivo* findings from Daehn *et al.* and Ebefors *et al.*^9^^,^^10^ Endothelial−podocyte crosstalk was probably mediated by increased expression of ET_A_R in glomerular endothelial cells, which implies increased ET-1 signaling; however, it is not an absolute indicator. ET-1 expression in glomeruli of patients with FSGS has been described before,^32^ yet its cellular source remains ambiguous. ET-1 is a small peptide produced by many cells, which makes immunohistochemistry results notoriously difficult to interpret. We have stained our patient cohort biopsy samples for ET-1 (data not shown), which resulted in a similar diffuse and nonspecific staining pattern and inconclusive results. It is also still unclear whether increased ET-1 signaling is due to (increased) ET-1 production by podocytes. ET_A_R expression in glomerular endothelial cells has rarely been reported and appears to be present only in specific pathological circumstances.^9^^,^^10^^,^^21^^,^^22^ Daehn *et al.* demonstrated that endothelial ET_A_R expression was increased in a murine model for FSGS, and also provided *in vitro* evidence for ET_A_R expression in glomerular endothelial cells.^9^ Supporting their findings, we show, for the first time, ET_A_R positivity in glomerular endothelial cells in biopsy samples of patients with FSGS. Further research is needed to investigate whether upregulation of ET_A_R expression in glomerular endothelial cells in FSGS is solely a reaction to increased ET-1 production or whether other factors might be involved, such as circulating serum factors. Our experimental setup did not allow us to investigate this mechanism.

Our results on ET_A_R expression in the glomerulus add to the existing knowledge of the endothelin system in FSGS and CKD progression.^9^^,^^11^^,^^15^^,^^16^^,^^33^ The current literature reports primarily a link between the endothelin system and the development of glomerulosclerosis in CKD or age-related processes, where it appears to be involved in a final common pathway of glomerular damage. Our data do not suggest that increased ET_A_R expression in FSGS is merely the result of age-related processes or CKD progression. Although we show an association between age and ET_A_R positivity, our cohort of patients with FSGS was significantly younger than the average age of individuals with age-related FSGS.^34^ In addition, we found no association of ET_A_R with estimated glomerular filtration rate (eGFR) at the time of biopsy. Furthermore, patients with good renal function also displayed increased endothelial ET_A_R expression. Finally, the association between ET_A_R positivity and podocyte injury in glomeruli without glomerulosclerosis, along with the mechanism described in experimental studies,^9^^,^^10^ implies that altered endothelial−podocyte crosstalk via the endothelin system is likely an early event in the development of FSGS.

It has been previously hypothesized that podocyte damage due to increased activation of the endothelin system in endothelial cells could be mediated via endothelial oxidative stress.^9^ We show elevated glomerular oxidative stress levels in a subset of patients with FSGS, which were associated with increased proteinuria and ET_A_R expression. Although it has been reported that oxidative stress contributes to podocyte damage, data on glomerular oxidative stress in human biopsy samples is limited.^9^, ^35^ *In* *vitro* experiments have shown that oxidative stress is increased in podocytes under FSGS-like conditions.^36^ Moreover, *in vivo* studies have shown that reduction of oxidative stress levels leads to a decrease in proteinuria. As the podocyte is thought to be the cell type mainly affected in FSGS, the effects of oxidative stress reduction in *in vivo* studies were attributed to lower oxidative stress in the podocyte.^17^, ^18^, ^19^, ^20^ In hindsight, the observed effects in these studies might partially be due to reduced oxidative stress in the glomerular endothelial cells. Here, we confirm that increased oxidative stress is associated with higher levels of proteinuria; however, it was not correlated with eGFR. As proteinuria often occurs prior to the development of renal function loss, this could explain the observed correlation with proteinuria, but not with eGFR. In the current study, we also show that 8-oxoG does originate from endothelial as well as podocytes. Yet, as we were not able to measure 8-oxoG levels in individual cell populations, their precise contribution to the pool of 8-oxoG−positive cells needs to be further investigated. Daehn *et al.* provided example images of a few patients with increased 8-oxoG levels in glomerular endothelial cells.^9^ In this study, we investigated a larger cohort and found an association between 8-oxoG levels and ET_A_R positivity, indicating that oxidative stress is related to ET-1 signaling in patients with FSGS. In contrast to the studies by Daehn *et al.* and Ebefors *et al.*, we found 8-oxoG to be increased in mitochondrial and primarily nuclear DNA, whereas 8-oxoG was exclusively located in mitochondria in their experimental model.^9^^,^^10^ Our finding of increased 8-oxoG in nuclear DNA is in line with previous *in vitro* data showing oxidative DNA damage to be present in podocyte nuclei during FSGS-like conditions.^36^ Differences in mitochondrial and nuclear oxidative DNA damage have been a topic of investigation. Mitochondrial DNA is more susceptible to ROS attack than nuclear DNA, because it is located in close proximity to the ROS generating electron transport chain.^37^^,^^38^ Nuclear DNA damage is caused by mitochondrial derived ROS as well, but a substantial portion of DNA damage can be caused by free radical production from other sources.^37^ Moreover, nuclear and mitochondrial oxidative damage lead to the activation of different repair and/or apoptosis-inducing pathways.^38^, ^39^, ^40^, ^41^ Thus, targets of mitochondrial and nuclear oxidative DNA damage in disease may be different.

The results of this study might have future therapeutic implications. ET_A_R antagonists have already emerged as promising therapies for treating chronic kidney disease.^11^ Several trials have shown the efficacy of ET_A_R blockers in treating diabetic nephropathy.^42^, ^43^, ^44^, ^45^, ^46^ Attempts to therapeutically block ET_A_R in FSGS have already been undertaken as well. The phase 2 DUET trial investigating the efficacy and safety of sparsentan, a dual ET_A_R and angiotensin receptor blocker, showed that sparsentan was more effective in reducing proteinuria than a single angiotensin receptor blocker in patients with FSGS.^47^ The follow-up phase 3 DUPLEX trial is currently being conducted and will provide us with additional information on the possibilities for endothelin receptor blocking in FSGS.^48^ Assessing ET_A_R positivity in renal biopsy samples of patients with FSGS may aid in the identification of those patients who might benefit from ET_A_R-blocking therapy.

In conclusion, we show that ET_A_R positivity in the glomerular endothelium is frequently present in patients with FSGS, and that it is associated with increased nephrin loss and 8-oxoG positivity. These data are in line with the previously proposed mechanism of altered podocyte−endothelial crosstalk via the endothelin system and oxidative stress in the development of FSGS, and suggest that this mechanism could play a role in human FSGS. Therefore, endothelin signaling in glomerular endothelial cells and its possible contribution to altered podocyte−endothelial interaction might be promising future therapeutic targets.

## Disclosure

All the authors declared no competing interests.

## Supplementary Material

Supplementary File (PDF)

**Table S1.** Demographic characteristics of control subjects

**Table S2.** Mean percentage of glomeruli with endothelin receptor A (ET_A_R) positive endothelium in males and females and among histological variants of FSGS

**Figure S1.** Blocking peptide experiment confirming the specificity of glomerular ET_A_R staining.

**Figure S2.** Examples of negative and positive controls for glomerular 8-oxo-guanine (8-oxoG) and ET_A_R immunofluorescence staining.

**Figure S3.** Examples of negative and positive controls for glomerular von Willebrand factor (vWF) and Wilms Tumor 1 (WT1) immunofluorescence staining.

**Figure S4.** Correlation between glomerular endothelial ET_A_R positivity and nephrin loss, excluding nephrin loss due to glomerular sclerotic lesions.

**Figure S5.** 8-oxoG−Positive area of individual glomeruli categorized by 8-oxoG−negative (green) and 8-oxoG−positive patients (red).

## References

[1] Jennette J.C., D'Agati V.D., Olson J.L. (2014). Heptinstall’s Pathology of the Kidney.

[2] Taneda S., Honda K., Ohno M. (2015). Podocyte and endothelial injury in focal segmental glomerulosclerosis: an ultrastructural analysis. Virchows Arch.

[3] Morita M., Mii A., Shimizu A. (2015). Glomerular endothelial cell injury and focal segmental glomerulosclerosis lesion in idiopathic membranous nephropathy. PLoS One.

[4] Buob D., Decambron M., Gnemmi V. (2016). Collapsing glomerulopathy is common in the setting of thrombotic microangiopathy of the native kidney. Kidney Int.

[5] Salvatore S.P., Reddi A.S., Chandran C.B. (2014). Collapsing glomerulopathy superimposed on diabetic nephropathy: insights into etiology of an under-recognized, severe pattern of glomerular injury. Nephrol Dial Transplant.

[6] Izzedine H., Escudier B., Lhomme C. (2014). Kidney diseases associated with anti-vascular endothelial growth factor (VEGF): an 8-year observational study at a single center. Medicine (Baltimore).

[7] Sun Y.B., Qu X., Zhang X. (2013). Glomerular endothelial cell injury and damage precedes that of podocytes in adriamycin-induced nephropathy. PLoS One.

[8] Kitamura H., Shimizu A., Masuda Y. (1998). Apoptosis in glomerular endothelial cells during the development of glomerulosclerosis in the remnant-kidney model. Exp Nephrol.

[9] Daehn I., Casalena G., Zhang T. (2014). Endothelial mitochondrial oxidative stress determines podocyte depletion in segmental glomerulosclerosis. J Clin Invest.

[10] Ebefors K., Wiener R.J., Yu L. (2019). Endothelin receptor-A mediates degradation of the glomerular endothelial surface layer via pathologic crosstalk between activated podocytes and glomerular endothelial cells. Kidney Int.

[11] Kohan D.E., Barton M. (2014). Endothelin and endothelin antagonists in chronic kidney disease. Kidney Int.

[12] Kuc R., Davenport A.P. (2004). Comparison of endothelin-A and endothelin-B receptor distribution visualized by radioligand binding versus immunocytochemical localization using subtype selective antisera. J Cardiovasc Pharmacol.

[13] Nambi P., Pullen M., Wu H.L. (1992). Identification of endothelin receptor subtypes in human renal cortex and medulla using subtype-selective ligands. Endocrinology.

[14] Benigni A., Buelli S., Kohan D.E. (2021). Endothelin-targeted new treatments for proteinuric and inflammatory glomerular diseases: focus on the added value to anti-renin-angiotensin system inhibition. Pediatr Nephrol.

[15] Chen H.C., Guh J.Y., Chang J.M. (2001). Plasma and urinary endothelin-1 in focal segmental glomerulosclerosis. J Clin Lab Anal.

[16] Ortmann J., Amann K., Brandes R.P. (2004). Role of podocytes for reversal of glomerulosclerosis and proteinuria in the aging kidney after endothelin inhibition. Hypertension.

[17] Diamond J.R., Bonventre J.V., Karnovsky M.J. (1986). A role for oxygen free radicals in aminonucleoside nephrosis. Kidney Int.

[18] Thakur V., Walker P.D., Shah S.V. (1988). Evidence suggesting a role for hydroxyl radical in puromycin aminonucleoside-induced proteinuria. Kidney Int.

[19] Ricardo S.D., Bertram J.F., Ryan G.B. (1994). Antioxidants protect podocyte foot processes in puromycin aminonucleoside-treated rats. J Am Soc Nephrol.

[20] Gwinner W., Landmesser U., Brandes R.P. (1997). Reactive oxygen species and antioxidant defense in puromycin aminonucleoside glomerulopathy. J Am Soc Nephrol.

[21] Qi H., Casalena G., Shi S. (2017). Glomerular endothelial mitochondrial dysfunction is essential and characteristic of diabetic kidney disease susceptibility. Diabetes.

[22] Niu J., Wu J., Li X. (2015). Association between endothelin-1/endothelin receptor A and inflammation in mouse kidneys following acute ischemia/reperfusion. Mol Med Rep.

[23] Tsuruya K., Furuichi M., Tominaga Y. (2003). Accumulation of 8-oxoguanine in the cellular DNA and the alteration of the OGG1 expression during ischemia-reperfusion injury in the rat kidney. DNA Repair (Amst).

[24] Ohno M., Oka S., Nakabeppu Y. (2009). Quantitative analysis of oxidized guanine, 8-oxoguanine, in mitochondrial DNA by immunofluorescence method. Methods Mol Biol.

[25] van de Lest N.A., Zandbergen M., Ijpelaar D.H.T. (2018). Nephrin loss can be used to predict remission and long-term renal outcome in patients with minimal change disease. Kidney Int Rep.

[26] Kanvah S., Joseph J., Schuster G.B. (2010). Oxidation of DNA: damage to nucleobases. Acc Chem Res.

[27] Hirsch R.E., Zukin R.S., Nagel R.L. (1980). Intrinsic fluorescence emission of intact oxy hemoglobins. Biochem Biophys Res Commun.

[28] Whittington N.C., Wray S. (2017). Suppression of red blood cell autofluorescence for immunocytochemistry on fixed embryonic mouse tissue. Curr Protoc Neurosci.

[29] Zhang Q., Zeng C., Fu Y. (2012). Biomarkers of endothelial dysfunction in patients with primary focal segmental glomerulosclerosis. Nephrology.

[30] Menon R., Otto E.A., Hoover P.J. (2020). Single cell transcriptomics identifies focal segmental glomerulosclerosis remission endothelial biomarker. JCI Insight.

[31] van de Lest N.A., Zandbergen M., Wolterbeek R. (2019). Glomerular C4d deposition can precede the development of focal segmental glomerulosclerosis. Kidney Int.

[32] Murer L., Zacchello G., Basso G. (1994). Immunohistochemical distribution of endothelin in biopsies of pediatric nephrotic syndrome. Am J Nephrol.

[33] Buelli S., Rosano L., Gagliardini E. (2014). Beta-arrestin-1 drives endothelin-1-mediated podocyte activation and sustains renal injury. J Am Soc Nephrol.

[34] Wiggins R.C. (2007). The spectrum of podocytopathies: a unifying view of glomerular diseases. Kidney Int.

[35] Kuo H.T., Kuo M.C., Chiu Y.W. (2005). Increased glomerular and extracellular malondialdehyde levels in patients and rats with focal segmental glomerulosclerosis. Eur J Clin Invest.

[36] Marshall C.B., Pippin J.W., Krofft R.D. (2006). Puromycin aminonucleoside induces oxidant-dependent DNA damage in podocytes in vitro and in vivo. Kidney Int.

[37] Phaniendra A., Jestadi D.B., Periyasamy L. (2015). Free radicals: properties, sources, targets, and their implication in various diseases. Indian J Clin Biochem.

[38] Kowalska M., Piekut T., Prendecki M. (2020). Mitochondrial and nuclear DNA oxidative damage in physiological and pathological aging. DNA Cell Biol.

[39] Bohr V.A., Dianov G.L. (1999). Oxidative DNA damage processing in nuclear and mitochondrial DNA. Biochimie.

[40] Paardekooper L.M., van Vroonhoven E., Ter Beest M. (2019). Radical stress is more cytotoxic in the nucleus than in other organelles. Int J Mol Sci.

[41] Oka S., Ohno M., Tsuchimoto D. (2008). Two distinct pathways of cell death triggered by oxidative damage to nuclear and mitochondrial DNAs. EMBO J.

[42] Mann J.F., Green D., Jamerson K. (2010). Avosentan for overt diabetic nephropathy. J Am Soc Nephrol.

[43] Dhaun N., MacIntyre I.M., Kerr D. (2011). Selective endothelin-A receptor antagonism reduces proteinuria, blood pressure, and arterial stiffness in chronic proteinuric kidney disease. Hypertension.

[44] Andress D.L., Coll B., Pritchett Y. (2012). Clinical efficacy of the selective endothelin A receptor antagonist, atrasentan, in patients with diabetes and chronic kidney disease (CKD). Life Sci.

[45] de Zeeuw D., Coll B., Andress D. (2014). The endothelin antagonist atrasentan lowers residual albuminuria in patients with type 2 diabetic nephropathy. J Am Soc Nephrol.

[46] Kohan D.E., Pritchett Y., Molitch M. (2011). Addition of atrasentan to renin-angiotensin system blockade reduces albuminuria in diabetic nephropathy. J Am Soc Nephrol.

[47] Trachtman H., Nelson P., Adler S. (2018). DUET: a phase 2 study evaluating the efficacy and safety of sparsentan in patients with FSGS. J Am Soc Nephrol.

[48] Study of sparsentan in patients with primary focal segmental glomerulosclerosis (FSGS). https://ClinicalTrials.gov/show/NCT03493685.

