# Supplementary material for: Endothelial Endothelin Receptor A Expression Is Associated With Podocyte Injury and Oxidative Stress in Patients With Focal Segmental Glomerulosclerosis
Source: Kidney Int Rep. 2021 Apr 22;6(7):1939–48. doi: 10.1016/j.ekir.2021.04.013 (PMC8258598; doi:10.1016/j.ekir.2021.04.013)
Supplement: Supplementary File (PDF) [file mmc1.pdf]

Supplementary material “Endothelial Endothelin Receptor A Expression Is Associated With Podocyte Injury and Oxidative Stress in Patients With Focal Segmental Glomerulosclerosis” by van de Lest *et al.*

Table of contents

Supplementary Table S1

Supplementary Table S2

Supplementary Figure S1

Supplementary Figure S2

Supplementary Figure S3

Supplementary Figure S4

Supplementary Figure S5

Supplementary Table S1: demographic characteristics of control subjects

	**Eurotransplant controls**
**Age, mean ± SD (years)**	58 ± 21
**Sex, male, n (%)**	4 (50)

SD: standard deviation

Supplementary Table S2: mean percentage of glomeruli with endothelin receptor A (ET_AR) positive endothelium in males and females and among histological variants of FSGS.

	**ET_AR positivity (mean percentage of glomeruli ± SD)**	***p*-value**
**Sex**		
Male	50 ± 24	0.65
Female	54 ± 30	
**FSGS variant**		0.66
NOS	52 ± 26	
Perihilar	20	
Cellular	46 ± 31	
Tip	52 ± 29	
Collapsing	66 ± 31	

ET_AR: endothelin receptor A FSGS: focal segmental glomerulosclerosis NOS: not otherwise specified SD: standard deviation

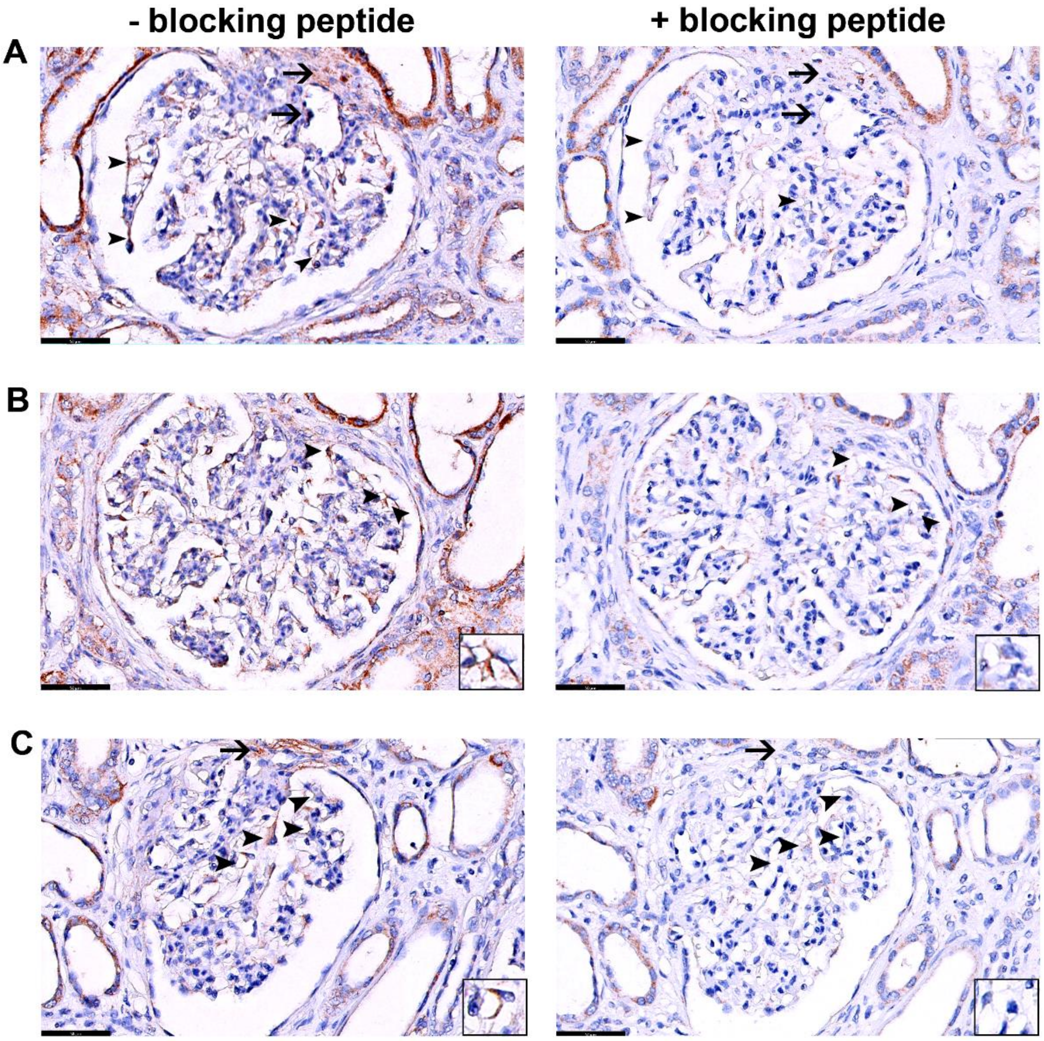

Supplementary Figure S1: blocking peptide experiment confirming the specificity of glomerular ET_AR staining. The specificity of the ET_AR primary antibody was assessed by using an ET_AR blocking peptide provided by the same manufacturer as the primary antibody. Five times excess blocking peptide to antibody weight was used. (A-C) ET_AR staining in glomeruli disappeared or was markedly reduced when the blocking peptide was added to the antibody solution (arrowheads; examples enlarged in insets). Also staining in the arterioles of the vascular pole, a known site for ET_AR positivity, disappeared after incubation with the ET_AR blocking peptide (arrows). Tubular staining was still present, but a significant reduction in staining was observed.

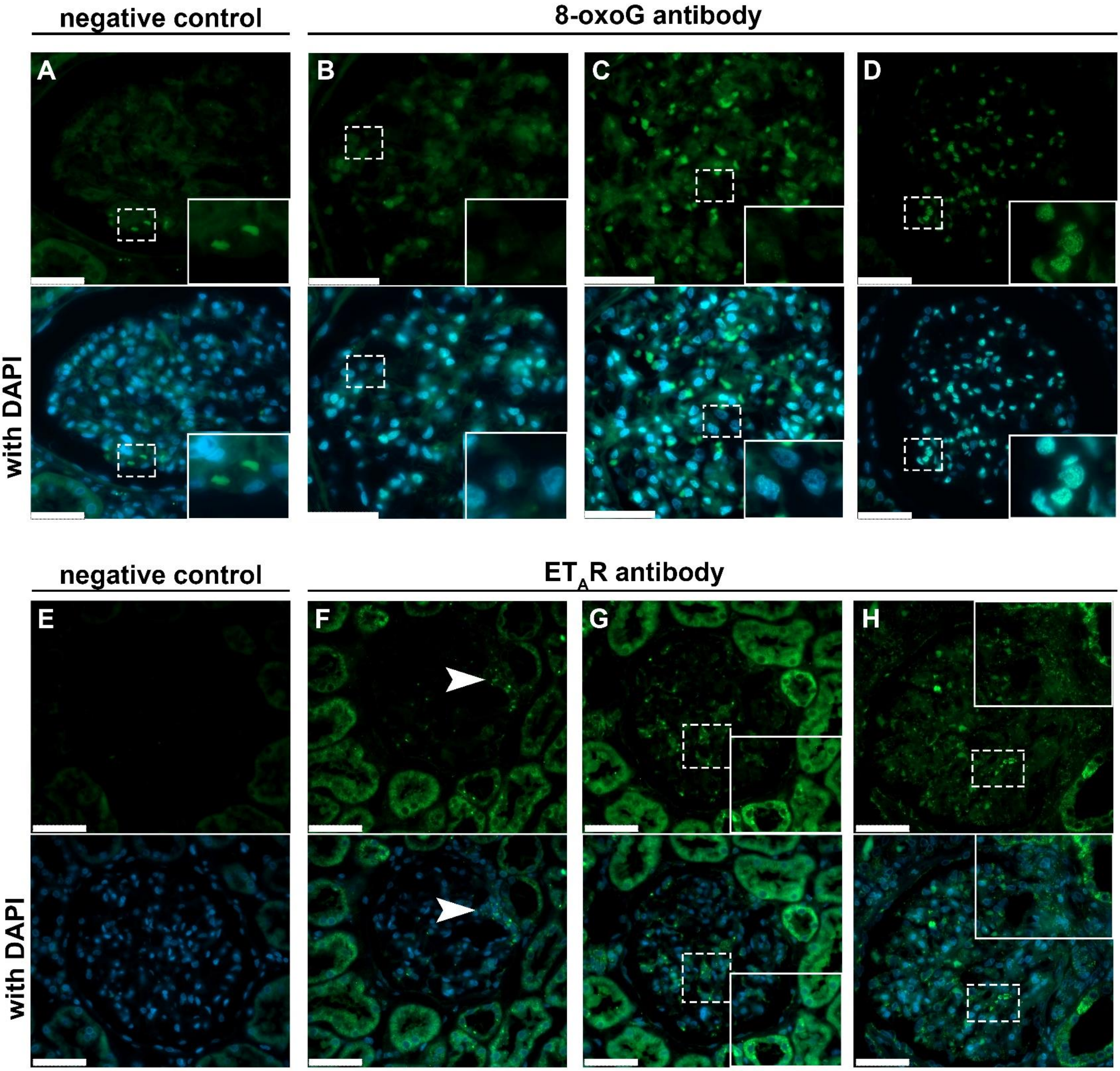

Supplementary Figure S2: **Examples of negative and positive controls for glomerular 8-oxo-guanine (8-oxoG) and ET_AR immunofluorescence staining.** (A-D) Representative images of negative and positive controls for the 8-oxoG staining. (A) Glomerulus of a kidney section incubated with mouse IgG2b fraction in the same concentration as the primary 8-oxoG antibody and a secondary donkey-anti-mouse antibody, thus serving as a negative control for nonspecific binding of mouse IgG2b and the secondary antibody. Apart from some background staining and unspecific staining of erythrocytes (enlarged in inset), the specific dotted 8-oxoG staining is completely absent. (B-D) Example glomeruli of kidney sections incubated with the primary 8-oxoG antibody and a secondary donkey-anti-mouse antibody. (B) Glomerulus incubated with the 8-oxoG

antibody but with hardly any 8-oxoG positive staining. The inset shows DAPI-positive nuclei that are negative for 8-oxoG. (C) Glomerulus with few 8-oxoG positive nuclei. The inset shows that most DAPI positive nuclei are negative for the specific dotted 8-oxoG staining. (D) Strong positive 8-oxoG staining in a larger number of nuclei. The typical dotted pattern is present (enlarged in inset). Still, some DAPI-positive nuclei are negative for 8-oxoG. (E-H) Negative and positive example images for ET_AR staining. (E) Glomerulus of a kidney section that has been incubated with rabbit polyclonal antibody fraction in the same concentration as the primary ET_AR antibody and a secondary goat-anti-rabbit antibody, thus serving as a negative control for nonspecific binding of rabbit immunoglobulins and the secondary antibody. Apart from some background staining, glomerular ET_AR staining is completely absent. (F-H) Example glomeruli of kidney sections incubated with the primary ET_AR antibody and a secondary goat-anti-rabbit antibody. (F) Some ET_AR positive staining is present at the glomerular vascular pole (arrowhead), but positive staining in the glomerulus is almost completely absent. (G-H) Positive controls for glomerular ET_AR staining, in which glomerular resident cells show positive staining for ET_AR (enlarged in insets). Scale bars represent 50µm.

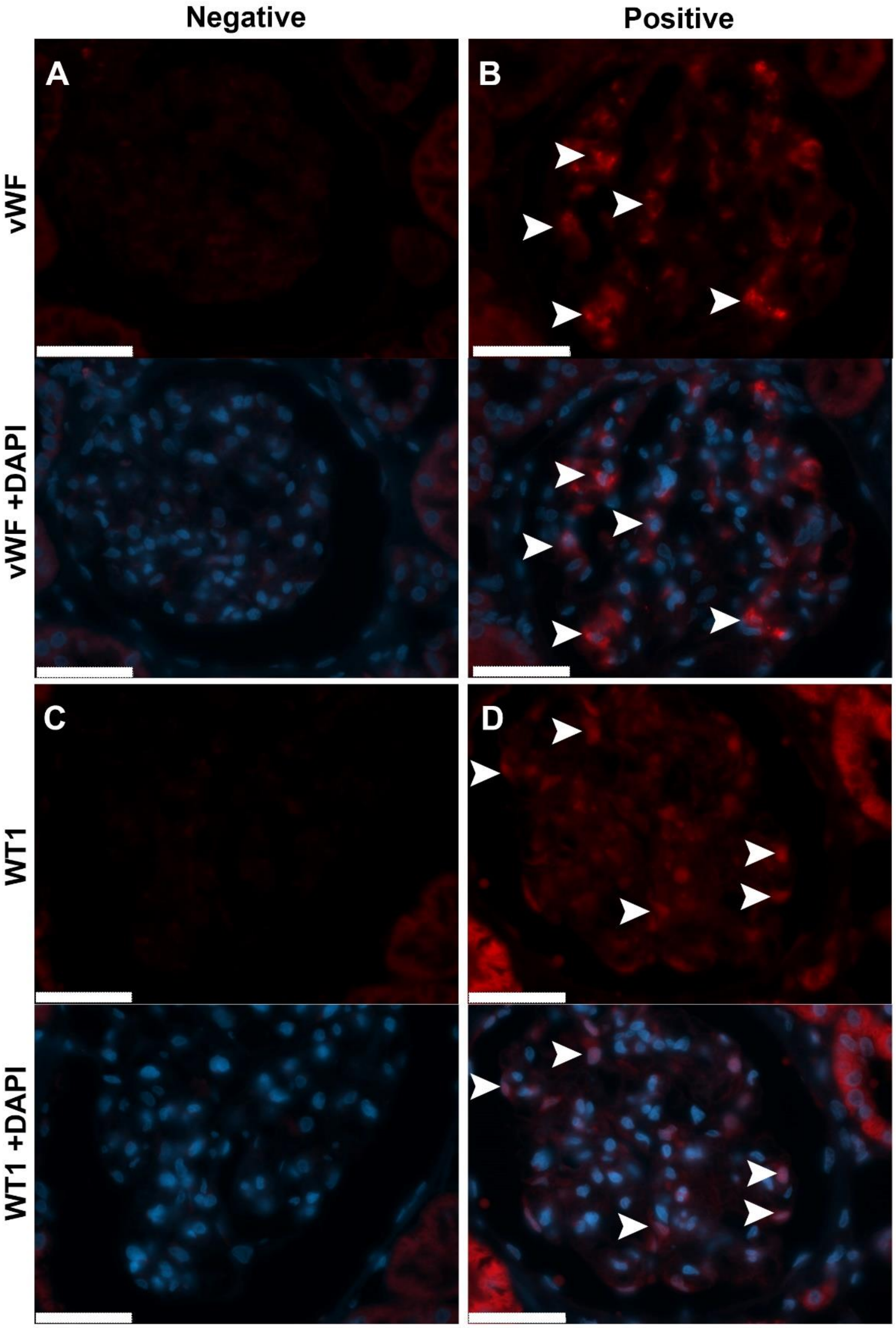

Supplementary Figure S3: Examples of negative and positive controls for glomerular von Willebrand factor (vWF) and Wilms Tumor 1 (WT1) immunofluorescence staining. (A-B) Negative and positive control for vWF immunofluorescence staining. (A) Glomerulus incubated with goat immunoglobulin fraction in the same concentration as the primary vWF antibody and a secondary donkey-anti-goat antibody, thus serving as a negative control for nonspecific binding of goat immunoglobulins and the secondary antibody. Apart from minor background staining, specific

staining is completely absent. (B) Positive vWF staining in glomerular endothelial cells (arrowheads). The section was treated with a primary goat anti-vWF polyclonal antibody. (C-D) Negative and positive control for WT1 immunofluorescence staining. (C) Glomerulus incubated with rabbit immunoglobulin fraction in the same concentration as the primary WT1 antibody and a secondary donkey-anti-rabbit antibody, thus serving as a negative control for nonspecific binding of rabbit immunoglobulins and the secondary antibody. Apart from minor background staining, specific staining is completely absent. (D) Positive WT1 staining in podocytes (arrowheads). The section was treated with a primary rabbit anti-WT1 polyclonal antibody. Although WT1 staining is not of the same intensity as vWF staining, clear colocalization of the red WT1 signal with DAPI-positive nuclei can be observed, as would be expected form WT1 staining. Scale bars represent 50µm.

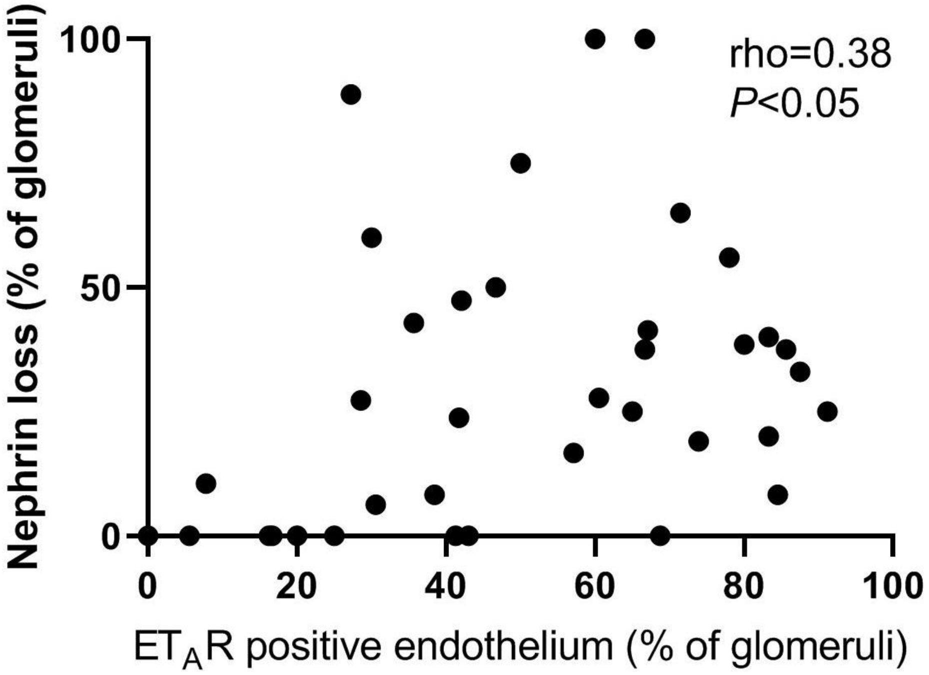

Supplementary Figure S4: correlation between glomerular endothelial ET_AR positivity and nephrin loss, excluding nephrin loss due to glomerular sclerotic lesions.

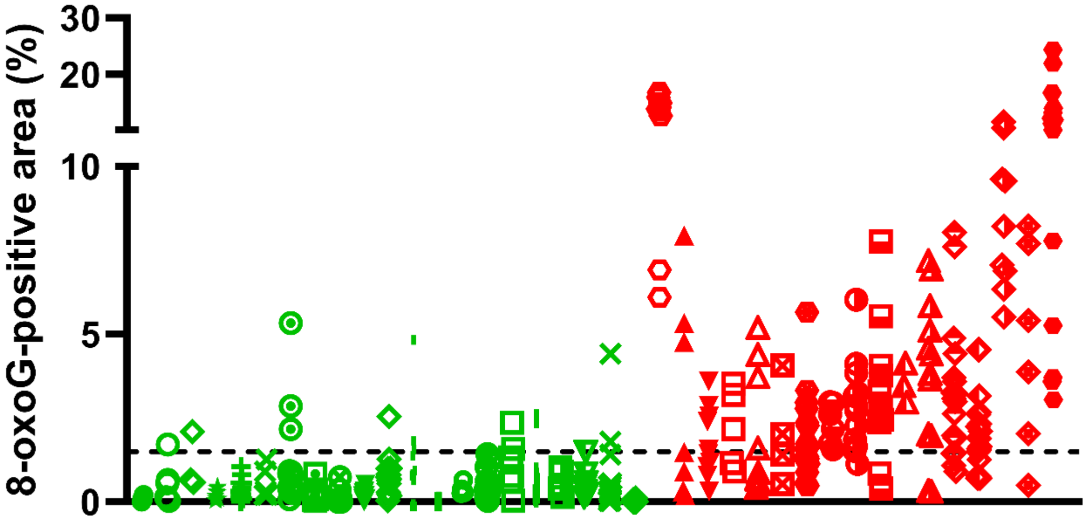

Supplementary Figure S5: 8-oxoG-positive area of individual glomeruli categorized by 8-oxoG-negative (green) and 8-oxoG-positive patients (red). In 8-oxoG-negative patients, defined as a mean 8-oxoG-positive area <1.5%, hardly any glomeruli passed the threshold of 1.5% positive area (dotted line). In 8-oxoG positive patients, most glomeruli showed 8-oxoG levels that were higher than 1.5%.
